# Biomimetic hydrogel micro-/nanofibers for *in situ* soft tissue repair and regeneration

**DOI:** 10.1016/j.bioactmat.2025.09.035

**Published:** 2025-09-30

**Authors:** Bingcheng Yi, Xiaoyu Wang, Jiajia Yu, Jiale Diao, Guangjun Wang, Shuo Li, Jiayi Bo, Xuemei Zhang, Chunling Zhang, Carlos F. Guimarães, Qihui Zhou, Rui L. Reis

**Affiliations:** a NHC Key Laboratory of Cardiopulmonary Rehabilitation and Functional Recovery (University of Health and Rehabilitation Sciences), Shandong Key Laboratory of Neurorehabilitation, Shandong Engineering Research Center for Tissue Rehabilitation Materials and Devices, School of Rehabilitation Sciences and Engineering, University of Health and Rehabilitation Sciences, Qingdao, 266113, China; b Department of Traditional Chinese Medicine, Qingdao Central Hospital, University of Health and Rehabilitation Sciences (Qingdao Central Hospital), Qingdao, 266042, China; c Department of Stomatology, The Affiliated Hospital of Qingdao University, Qingdao University, Qingdao, 266003, China; d School of Clinical Medicine, Shandong Second Medical University, Weifang, 261053, China; e Department of Hepatobiliary, Thyroid, Breast and Burns Surgery, Qingdao West Coast New Area Central Hospital, Qingdao, 266555, China; f 3B's Research Group-Research Institute on Biomaterials, Biodegradables and Biomimetics, University of Minho, Headquarters of the European Institute of Excellence on Tissue Engineering and Regenerative Medicine, Guimarães, 4805-017, Portugal; g ICVS/3B's – Portuguese Government Associate Laboratory, University of Minho, Guimarães, Braga, Portugal

**Keywords:** Hydrogel micro-/nanofibers, *In situ* soft tissue regeneration, Biomimetic features, Cell responses, Material design

## Abstract

To effectively harness the regenerative potential of the body and orchestrate cellular responses for *in situ* tissue repair, the design of biomaterials requires careful consideration of precise modulation of biophysical and biochemical cues. This is essential to maximize the guidance of endogenous cell responses at the injury site. Hydrogel micro-/nanofibers, which integrate the benefits of hydrogel biomaterials and micro-/nanofiber architectures into a unified scaffold, have emerged as innovative biomimetic substrates that closely mimic the physiological characteristics of native extracellular matrix. These substrates exhibit tissue-like polymer networks, rapid responsiveness to microenvironmental changes, and permeability to essential nutrients and oxygen. Their biomimetic attributes facilitate cell recruitment and diffusion for angiogenesis, nutrient diffusion for cell self-renewal, and cell-material interactions for matrix remodeling, thus effectively harnessing the regenerative capacity of the body for tissue-specific regeneration. This review offers an overview of recent advances in hydrogel micro-/nanofiber design and their applications in *in situ* soft tissue engineering, focusing on: I) the concept and biomimetic characteristics of hydrogel micro-/nanofibers; II) current fabrication strategies, including material selection and preparation methods; and III) research progress in employing hydrogel micro-/nanofibers for *in situ* soft tissue regeneration, particularly in nerve, skin, cardiovascular, and skeletal muscle tissues. Overall, leveraging the body's regenerative potential through biomimetic hydrogel micro-/nanofibers represents an effective and promising approach for restoring damaged tissues. Additionally, this review provides valuable insights to foster interdisciplinary knowledge exchange and enables the development of prognostic markers for the next generation of hydrogel micro-/nanofibers to accelerate soft tissue regeneration.

## Highlights

• The **properties and advantages** of hydrogel micro-/nanofibers for mimicking natural extracellular matrices are summarized.
• C**urrent advancements** in utilizing hydrogel micro-/nanofibers for *in situ* tissue repair and regeneration are introduced.
• **Comparative analysis** of fabrication techniques, including electrospinning and microfluidics, for specific tissue applications is conducted.
• **Applications across tissues like** nerves, skin, cardiovascular, and muscles, demonstrate their versatility in regeneration.
• **Key challenges and limitations** in material properties, mechanical strength, and clinical translation are discussed.

## Graphical abstract

Applications of hydrogel micro-/nanofibers for tissue repair and regeneration.

**Figure:**
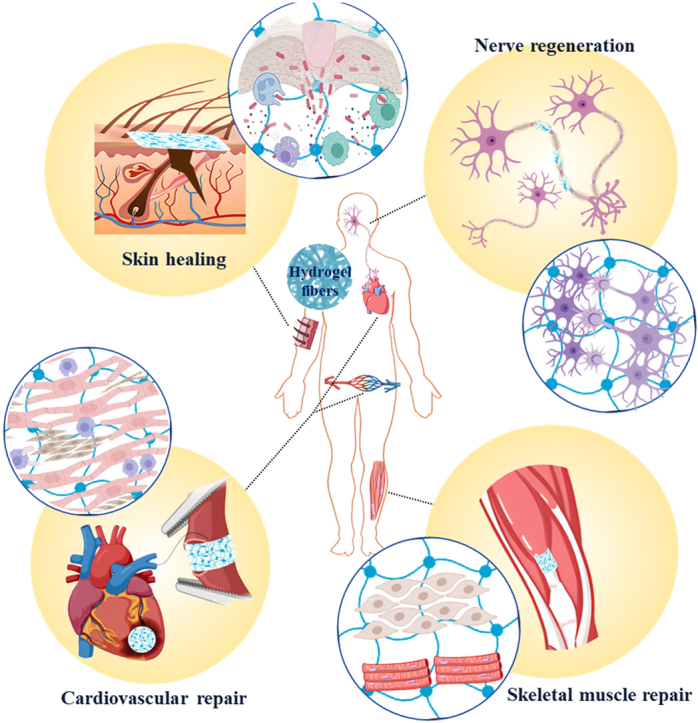

**Table:** Abbreviations

**2D**	Two-dimensional	**MAP-2**	Microtubule associated protein 2
**3D**	Three-dimensional	**MeGG**	Methacrylated gellan gum
**ACG**	Aligned chitosan fiber hydrogel	**MHC**	Major histocompatibility complex
**ADH**	Adipic acid dihydrazide	**MSC**	Mesenchymal stem cell
**AFG/fSAP**	Aligned fibrin/functionalized self-assembling peptide	**NCAM**	Neural cell adhesion molecule
**ASC**	Adipose-derived stem cell	**NGF**	Nerve growth factor
**BDNF**	Brain-derived neurotrophic factor	**NSC**	Neural stem cell
**BSA**	Bovine serum albumin	**PAA**	Polyacrylic acid
**CM**	Cardiomyocyte	**PAN**	Polyacrylonitrile
**CNT**	Carbon nanotube	**PC**	Phycocyanin
**CS**	Chitosan	**PEG**	Polyethylene glycol
**DAMP**	Danger-associated molecular pattern	**PEGDA**	Poly (ethylene glycol) diacrylate
**DAPI**	4′,6-diamidino-2-phenylindole	**PEO**	Polyethylene oxide
**DCMC**	Dialdehyde carboxymethyl cellulose	**PL**	Platelet lysate
**ECM**	Extracellular matrix	**PRP**	Platelet-rich plasma
**EC**	Endothelial cell	**PVA**	Polyvinyl alcohol
**FGL**	AcN-EVYVVAENQQGKSKA-CONH_2_	**RADA_16_**	AcN-RADARADARADARADA-CONH_2_
**GA**	Gallic acid	**RADA_16_-FGL**	AcN-RADARADARADARADAGGEVYVVAENQQGKSKA-CONH_2_
**GelMA**	Gelatin methacryloyl	**RC-33**	1-[3-(1,10-biphenyl)-4-yl]butylpiperidine
**GFAP**	Glial fibrillary acidic protein	**RGI**	RGIDKRHWNSQ
**HA**	Hyaluronic acid	**SAM**	Self-assembled monolayer
**HE**	Hematoxylin-eosin	**SCI**	Spinal cord injury
**HF**	Hydrogel micro-/nanofiber	**SMC**	Smooth muscle cell
**hMVEC**	Human microvascular endothelial cell	**sNAG**	Shorten poly-N-acetyl glucosamine
**HUVEC**	Human umbilical vein endothelial cell	**TEM**	Transmission electron microscope
**iPSC**	Induced pluripotent stem cell	**VEGF**	Vascular endothelial growth factor
**QK**	KLTWQELYQLKYKGI	**VML**	Volumetric muscle loss

## 1 Introduction

All organisms, including humans, possess some degree of self-healing and regenerative capability, which relies on the coordinated actions of substrates and cell lineages—a process known as cell-matrix interaction [1,2]. Efficient tissue regeneration necessitates tight coordination of numerous cellular events, involving cell migration, proliferation, matrix deposition and remodeling, angiogenesis, and homeostasis [[3], [4], [5]]. Conversely, the remodeled ECM provides a complex environment comprising biochemical composition and biophysical cues to modulate cell renewal, growth, and differentiation [6,7]. This thereby fabricates satisfactory tissue repair. While minor injuries can undergo autonomous healing in healthy individuals, more extensive injuries or the presence of chronic diseases frequently compromise the regenerative process through mechanisms that remain incompletely elucidated [8]. This results in the disordered regeneration and function reconstruction of damaged tissues. As an emerging subset of regenerative medicine, *in situ* tissue engineering can leverage the physicochemical and biochemical properties of biomaterials to directly guide host cell growth and harness the innate regenerative potential of the mammalian body [9]. Hence, it has garnered considerable attention for repairing these larger injuries or reconstructing complex tissues. For example, engineering the biomaterials through replicating the properties of the targeted ECM can offer a structural framework to facilitate the attachment, proliferation, and growth of host stem and progenitor cells. By incorporating bioactive cues, these biomaterials can further precisely modulate cellular responses and drive their differentiation into tissue-specific cell types through cell-matrix interactions, ultimately accelerating tissue repair [1,6].

Hydrogels mimic the high water content and mechanical viscoelasticity of the ECM. Previous studies have demonstrated that these biomimetic characteristics of hydrogel are advantageous for cell metabolism and matrix remodeling [10,11], while simultaneously maintaining the permeability of oxygen and essential nutrients [[11], [12], [13], [14]]. Nevertheless, hydrogels fail to replicate the nanoscale filamentous structure of native ECM accurately. In contrast, micro-/nanofibers, a broad categorical term referring to ultrafine fibers with diameters on the micrometer or nanometer scale, exhibit tissue-like polymer networks and provide the large specific surface areas for the enhancement of cell growth, infiltration, and cell-matrix signaling [15]. Regrettably, fibrous scaffolds typically lack the biomimetic microenvironment characterized by high water content for cell metabolism. To address these limitations, integrating the advantages of hydrogels with those of nanofibers to develop hydrogel micro/nanofibers (HFs) has recently emerged as an approach for constructing novel biomimetic platforms [[16], [17], [18]]. HFs refer to fibrous materials whose diameters range from microns to nanoscale, where each fiber intrinsically exhibits hydrogel-like properties (e.g., high water content, swellability, and softness), and can be further crosslinked among each other into larger-scale constructs for application in tissue repair. A growing body of evidence confirms that HFs enable precise control of endogenous cell behavior, enhance cell self-renewal, and improve cell-material interactions. For example, the plasticity and ECM-mimetic architecture of HFs allow the development of cellular tractions analogous to those within the native ECM. This capability effectively transmits critical biophysical and biochemical cues to promote bidirectional cell-material interactions [19], thereby enhancing cellular mechanotransduction, metabolic activity, and biological function.

Furthermore, intelligent responsive HFs can be engineered to emulate the stimulus-response mechanisms of biological systems. This biomimicry enhances the matrix's ability to sense and respond to environmental changes [20], allowing for the precise regulation of endogenous cell behavior [21]. However, their development has been hindered by the challenge of balancing mechanical properties with biological functionality. For example, HFs typically exhibit high viscoelasticity, which often results in relatively weak mechanical strength. Despite this limitation, the unique mechanical properties of HFs make them promising candidates for the repair of soft tissues, such as nerve, skin, cardiovascular system, and skeletal muscle, where dynamic viscoelasticity and mechanical biocompatibility are crucial.

In recent years, growing research interest in HFs has led to several reviews summarizing their development within specific domains [22]. Existing reports have primarily focused on applications in biointerfacing technology [23], wound healing [24], biomedical devices [25] (e.g., drug delivery and bioadhesives), and sensors [20]. These reviews have provided excellent summaries of the current state of development and offered forward-looking perspectives for readers. In contrast, this review begins with raw materials and systematically examines key considerations in the structural design and material preparation strategies for fabricating HFs. It further explores their advancing applications in soft tissue repair. We anticipate that a comprehensive analysis of current progress in HFs for soft tissue regeneration will enhance researchers’ understanding of this field and guide the design of next-generation HFs, potentially enabling bionic functionalities that closely mimic those of native human tissues.

**Fig. 1:**
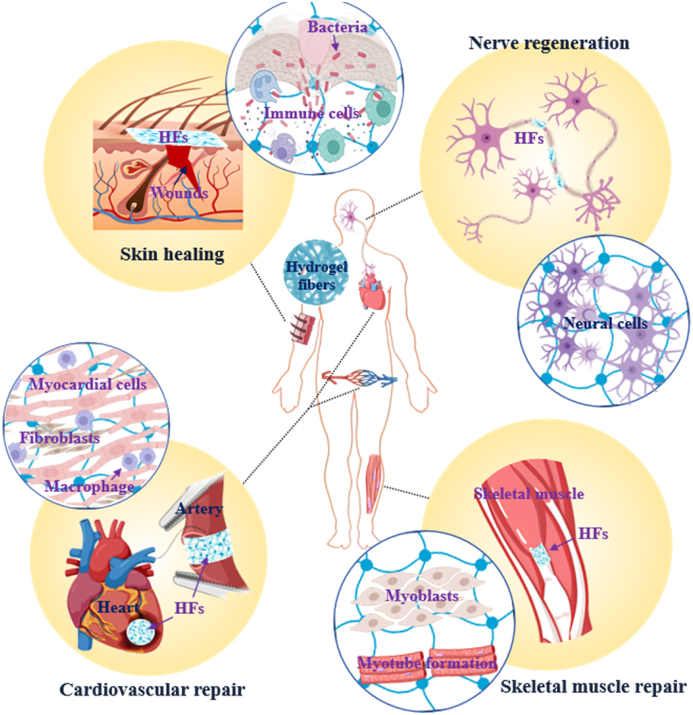
Applications of HFs for soft tissue repair and regeneration (Created with BioRender.com and obtained its permission).

Herein, this review provides an overview of recent advancements in biomimetic HFs for *in situ* repair and regeneration of soft tissues (Fig. 1). Initially, the biomimetic attributes of HFs are briefly described, encompassing the conceptual framework and development history of these biomaterials, as well as the impact of their biomimetic characteristics on cellular responses and tissue repair. Afterward, current strategies for fabricating HFs are summarized, with an emphasis on the selection of appropriate raw biomaterials and the fabrication methodologies developed to generate the substrates. Finally, given the unique mechanical compliance of HFs, this review summarizes the research progress in employing HFs for *in situ* regeneration and repair of specific soft tissues, including nerves, skin, cardiovascular tissues, and skeletal muscles, and discusses potential avenues for future research.

## 2 Biomimetic characteristics of HFs

### 2.1 Origin and conceptual framework of HFs

Natural ECM is a hydrated and dynamic 3D polymeric network, consisting of fibrous proteins (e.g., collagen and fibronectin) and proteoglycans (e.g., perlecan and biglycan). Fibrous proteins form interconnected networks to provide the structural support of the ECM, while proteoglycans fill the extracellular interstitial space [26]. Cells naturally exist in the 3D microenvironment. They interact with the ECM through pulling (*via* actin-based contraction coupled to the ECM with integrin-based adhesion) and pushing (*via* actin polymerization and depolymerization) the substrate, and then sense the extracellular signals, regulate intracellular processes, mediate cell fate, and ultimately control ECM remodeling [27,28]. This fibrous organization of ECM, in turn, promotes long-distance intercellular communication. That is, a contraction-induced strain within one cell can propagate along the fibers to a distant cell, subsequently being translated into a biochemical signal to regulate various cellular functions, including contraction, migration, and activation. These interactions of cell-cell and cell-matrix are crucial for tissue homeostasis and disease pathogenesis [6,12]. They govern the reconstruction and repair of damaged tissues in the physiological state after minor injuries. For example, in skin injuries, the body secretes growth factors to activate fibroblast proliferation and extracellularly stimulate ECM synthesis and aggregation [3]. Such formed ECM then supports the growth of newly proliferating cells and promotes cell migration versus proliferation for tissue regeneration [29]. However, once the damage exceeds the body's inherent capacity for self-repair, intervention with biomaterials is vital to provide structural support for cell growth and control cell behaviors to regulate ECM remodeling. In this circumstance, biomaterials rapidly recruit host cells from surrounding tissues and regulate their functions by utilizing their physicochemical properties (e.g., stiffness, composition, and topography). Cells, in turn, degrade and remodel the materials by secreting matrix-degrading enzymes and constructing new ECM, ultimately achieving tissue regeneration through the dynamic cell-matrix interactions [30]. Given that the biomaterial-based intervention represents a promising strategy for tissue repair, the current research efforts focus on optimizing the physicochemical properties of biomaterials to enhance cell-matrix interactions through faithfully recapitulating the natural ECM.

Hydrogels, 3D water-swollen polymer networks, are appealing as tissue-engineered scaffolds as they provide a similar aqueous environment to the natural ECM (typically >70 % water content) and feature tunable physicochemical properties (e.g., biocompatibility, softness, and chemical signaling) tailored to specific conditions [[31], [32], [33], [34]]. Such adaptability allows hydrogels to closely mimic the cellular microenvironment, which enhances cell survival, encapsulation, and tissue regeneration [35,36]. Consequently, hydrogels with tissue-adhesive [24,37], self-healing [38,39], injectable [40], conductive [41], or smart responsive properties [42] have been developed for applications in skin, bone, cartilage, cardiovascular, and nerve regeneration. However, conventional crosslinked hydrogels struggle to manage stress concentration and exhibit poor mechanical properties [24]. Their dense honeycomb mesh-like structure restricts cell-matrix interactions and impedes biomaterial-induced tissue regeneration [26,43]. In contrast, fibrous substrates, characterized by tissue-like polymer networks, allow long-distance intercellular communication and provide abundant adhesive sites and the requisite softness for cell-matrix interactions [44,45]. This environment supports mechanotransduction for cell responses and matrix remodeling. Nevertheless, micro-/nanofibers often lack sufficient moisture content and appropriate viscoelasticity, limiting cell metabolism [46]. In this context, a novel hydrogel with a nanofibrous structure, termed HFs, has recently been explored and formed by integrating fiber formation technology and crosslinking strategies. This approach combines the merits of hydrogels (e.g., high water content, high viscoelasticity, and rapid environmental responsiveness) and micro-/nanofibers (e.g., high specific surface area, ease of weaving, and permeability to vital nutrients and oxygen) into a single scaffold, offering enhanced potential for clinical applications [30,31].

### 2.2 Biomimetic properties of HFs

**Fig. 2:**
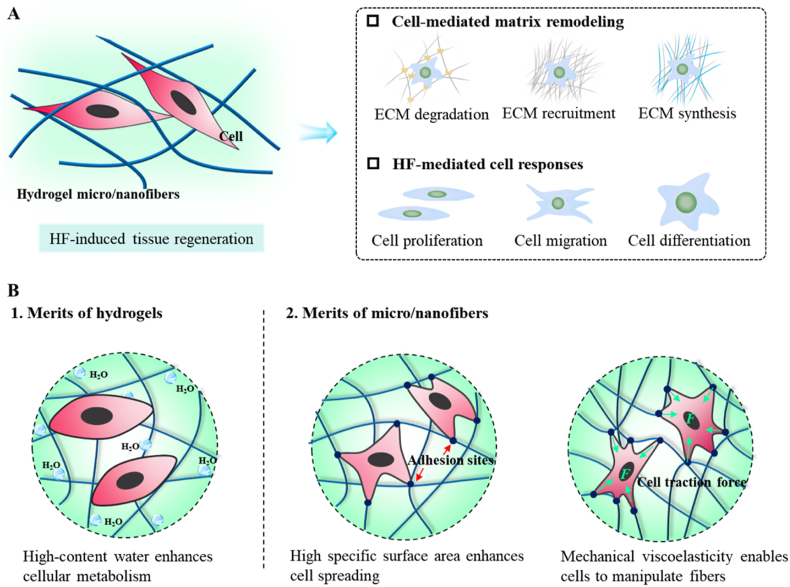
Biomimetic properties of HFs for tissue regeneration: (A) Cell-matrix interactions; (B) Merits of HFs for modulating cell behaviors.

When HFs are implanted as substrates to modulate tissue repair and regeneration *in situ*, the host cells can be recruited to adhere to the biomaterials and form focal adhesions, recognizing their biochemical signals and the physical cues, such as surface pattern, stiffness, contractility, and dimensionality [45]. This complex array of extracellular signals is then transduced into biochemical signals and initiates intracellular signaling cascades, ultimately activating cell responses, including cell migration, proliferation, and differentiation (Fig. 2A). Recent studies have verified that the ultrafine structure of HFs can act in concert with their high moisture content and high viscoelasticity to promote cell adhesion and growth [47], facilitate the macrophage infiltration to induce vascularization [48], and increase nutrient permeability to enhance cell self-renewal [49] (Fig. 2B). These findings suggest that the biomimetic properties of HFs primarily arise from the combination of hydrogels and micro-/nanofibers.

Hydrogels demonstrate excellent biocompatibility with native tissues. This is attributed to their physiological resemblance, including high moisture content, rapid environmental responsiveness, tunable viscoelasticity, and permeability to oxygen and essential nutrients. For example, the high moisture content of hydrogels benefits cell metabolism and growth, while their high porosity (up to several microns) facilitates the transport and dissolution of cellular nutrients and waste through the substrates [31]. The rapid responses to environmental stimuli allow hydrogels to be designed for the controlled behaviors of cells or the release of bioactive molecules in response to stimuli like temperature, pH, or light [50]. Their tunable viscoelasticity, which undergoes time-varying square creep or deformation in response to external stress/load, allows cells to overcome limitations in size and morphology changes through diffusion and migration [43]. Such mechanical biocompatibility improves cell-matrix interactions for tissue remodeling [51]. In the case of micro-/nanofibers, their ECM-like structure is mainly determined by factors such as fiber diameter, fiber arrangement, and biomaterial composition. It has been reported that the decrease in fiber diameter enables the increased surface area to adsorb higher proteins, and expose their cryptic adhesion sites by changing conformation or directly providing more adhesion sites for receptors on the cell membrane [52]. This favors cell adhesion and ECM proteins (e.g., collagen and elastin) secretion [53]. Recognizing the fact that micro-/nanofibers guide cell spreading and growth through contact guidance, significant efforts have been made to construct the structure-aligned fibrous scaffolds to replicate the inherent anisotropic architecture of the vascular tissue [54,55]. Moreover, their elevated specific surface area enables the binding of biomolecules or drugs into micro-/nanofibers, thereby enhancing their biofunctions and leading to their widespread applications in tissue regeneration, such as vessel [55], nerve [56], and bone [57]. Overall, HFs constitute an advanced class of biomaterials that integrate the advantageous features of hydrogels with the structural benefits of fibers. In comparison to other biomaterials, HFs offer several key advantages for promoting effective *in-situ* tissue regeneration: 1) Their biomimetic architecture, recapitulating the topographic and dimensional features of the native ECM, provides a contact-guided effect conducive to cell adhesion, migration, and tissue organization [58]. These properties often lack in bulk hydrogels or smooth-surface materials; 2) The fibrous network enhances cellular mechanotransduction by enabling cells to exert natural traction forces [19]. This improves cell differentiation and function compared to many non-fibrous materials where mechanical cues are less effectively presented; 3) The highly porous and interconnected structure of HFs facilitates efficient nutrient diffusion, metabolic waste removal, and vascular ingrowth [20]. This overcomes the diffusion limitations associated with dense hydrogel systems, preventing core necrosis within the implant; 4) The mechanical strength and elasticity can be precisely tailored to match specific tissue requirements through adjusting fiber density, alignment, and crosslinking density [59]. This tunability offers a significant advantage over rigid pre-formed scaffolds that cannot conform to irregular defects; 5) The high surface-area-to-volume ratio of fibrous networks allows for facile biofunctionalization *via* immobilization of bioactive molecules [22]. This enables sustained and localized signaling to cells through a more efficient strategy than bulk blending of cues; 6) Owing to their hydrogel features, many HFs may be engineered as injectable or sprayable formulations, permitting minimally invasive delivery and conformal filling of complex defects. This combines patient-friendly implantation with the ECM-mimetic advantages of a fibrous scaffold.

## 3 Design strategies and development of HFs

Generally, the structural characteristics of hydrogels dictate the challenges associated with HF preparation. This is primarily due to the inadequate viscoelasticity of their precursor solutions, consisting of low molecular weight oligomers and monomers. Besides, attributing to constraints imposed by the cross-linked structure, the formed hydrogels often struggle to endure tensile deformation during fiber processing [24]. Therefore, the construction of suitable HFs with desired hydrogel properties and fibrous functionality necessitates meticulous consideration of several critical factors, encompassing both early-stage raw material selection and late-stage process preparation. The choice of appropriate raw materials is crucial to the successful preparation of HFs, necessitating compounds capable of cross-linking to form hydrogels while also permitting intermolecular entanglement to facilitate fiber generation. Such materials can be either natural or synthetic, each possessing unique advantages and limitations. For example, natural materials (e.g., collagen, gelatin, and HA) are generally regarded as safe alternatives for cell support; however, they often exhibit batch-to-batch variability and possess limited mechanical strength [60]. Conversely, synthetic materials like PEG and PVA show tunable mechanical properties and degradation rates but may lack inherent bioactivity. Additionally, the fabrication method dictates the type and properties of HFs, which primarily involves two steps: 1) micro-/nanofiber formation and 2) hydrogel generation. This section explores the recent studies on material selection and preparation methods for the fabrication of HFs.

### 3.1 Material consideration for HFs

HF preparation necessitates that the raw materials exhibit the capability to form hydrogels and generate micro/nanofibers. Hydrogels are 3D cross-linked polymer networks that contain hydrophobic and hydrophilic residues [61]. The hydrophilic residues absorb and retain water molecules within the network, while the hydrophobic residues contribute to the expansion upon water contact [62]. Cross-linking, as a stabilization process in polymer chemistry, contributes to the formation of stable network structures and maintains the multidimensional extension of polymeric chains in hydrogels. In this context, it is speculated that materials for preparing HFs should exhibit a significant number of hydrophilic groups on the main/side chain of the molecules and provide adequate cross-linking sites for network formation. Hydrogels can be categorized into two primary groups based on the nature of their cross-linking junctions: chemically cross-linked and physically cross-linked [63]. Chemically cross-linked hydrogels possess permanent junctions with covalent bonds among polymer chains.

**Fig. 3:**
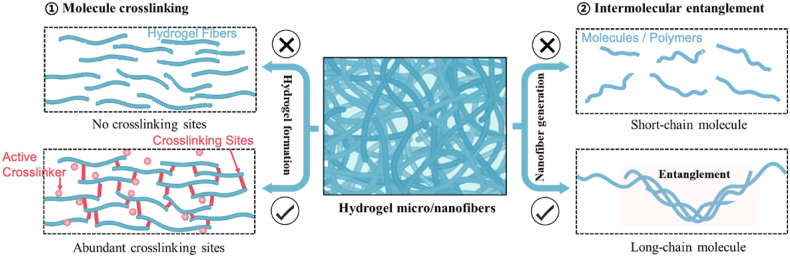
Consideration of raw materials for HF preparation: abundant cross-linking sites and intermolecular entanglement.

In contrast, physically cross-linked hydrogels maintain their structure through non-covalent interactions such as electrostatic forces, hydrogen bonds, ionic interactions, and hydrophobic interactions. Based on this, almost all water-soluble or hydrophilic polymers are hypothesized to possess the ability to form hydrogels through proper chemical or physical cross-linking methods [32]. Regarding fiber formation, the choice of polymer is crucial. The polymer should exhibit good solubility in a suitable solvent, enabling the formation of a stable solution or dispersion. Besides, the polymer molecules in the solution should be able to entangle for fiber generation [64]. In this regard, materials for HFs should possess adequate water-soluble or hydrophilic groups as cross-linking sites for hydrogel formation. Meanwhile, they should exhibit good solubility in a suitable solvent, allowing for molecular entanglement and subsequent fiber generation (Fig. 3).

Recently, various biomaterials have been developed for the formation of HFs, including natural and synthetic polymers (Table 1). Natural polymers, known for their biocompatibility, biodegradability, bioactivity, and low immunogenicity, have been extensively utilized in the development of HFs for repairing and regenerating damaged tissues, such as nerve [65], skin [24], skeletal muscle [66], and blood vessels [67]. These polymers primarily consist of polysaccharides and proteins/peptides. In comparison to proteins/peptides, polysaccharides are typically stable and do not denature under heat exposure [68]. Besides, polysaccharides exhibit high chemical reactivity, chirality, chelation, and adsorption capacity owing to their multifunctional groups, thus allowing for facile modification through chemical and biochemical methods [69]. For example, catechol-tethered alginate was employed to fabricate 3D hydrogel fibrous constructs *via* wet-spinning for cell encapsulation. This structurally robust scaffold showed the capability to sustain a high cell density over a 24-h culture period, highlighting its potential for use in cell transplantation and tissue regeneration [70]. CS HFs with directional alignment were prepared by combining electrospinning and mechanical stretching techniques. Through grafting with RGI and QK, which mimic BDNF and VEGF, respectively, this scaffold markedly enhanced nerve regeneration and facilitated vascular penetration [65].

**Table 1:** Biomaterials for the preparation of HFs for *in situ* tissue repair and regeneration.

Materials		Advantages	Disadvantages	Scaffolds	Preparation-methods	Applications	Ref.
**Natural polymers**	**Polysaccharide**	Bioactivity; Low immunogenicity; Metabolic adaptability.	Poor mechanical strength; Risk of pathogen transmission; Batch-to-batch variability.	Sodium alginate	Electrospinning and free radical polymerization;	Drug transportation;	[47]
					Wet spinning and calcium ion crosslinking;	Biomedical fields; Mechanical and medicinal properties;	[78,79]
					Microfluidic and radical polymerization;	Artificial muscle; Regenerative medicine.	[80,81]
					Electrospinning and calcium ion crosslinking.	Cell culture	[80]
				HA	Electrospinning and enzymatic catalysis	Cell encapsulation and tissue regeneration; Wound healing	[82,83]
				CS	Electrospinning and electrostatic interaction;	Nerve regeneration; Drug delivery.	[57,84]
					Molecular self-assembly.	Nerve regeneration	[83]
	**Protein**			Gelatin	Electrospinning and photopolymerization;	Skin repair; Bone regeneration.	[82,83]
					Wet spinning and ionic crosslinking.	Engineering tissue scaffolds	[85]
				GelMA	Electrospinning and photopolymerization	Nerve regeneration; Skin tissue engineering.	[86,87]
				Silk fibroin	Electrospinning and thermal polymerization;	Tendon tissue engineering	[86]
					Dry–wet electrospinning and photocrosslinking	Peripheral nerve tissue engineering	[87]
**Synthetic polymers**		High repeatability; Large-scale production; Tunable properties, including stiffness and degradation.	Bioinertness; Potential cytotoxicity of degradation product.	PVA	Electrospinning and glutaraldehyde (Glu) crosslinking;	Cell culture and tissue engineering	[88]
					Electrospinning and physical/chemical crosslinking	Skin tissue engineering.	[89]
				PAA	Microfluidic spinning and thermo-crosslinking;	Reversible pH-responsive property	[90]
				PEG	Electrospinning and chemical crosslinking	Skin tissue engineering	[91]
				PEGDA	Electrospinning and ionic crosslinking;	Tissue regeneration	[66]
					Electrospinning and catalytic polymerization	Wound healing	[67]

In contrast to polysaccharides, proteins/peptides demonstrate versatility, flexibility, metabolic adaptability, and the capacity to mimic the native ECM, rendering them well-suited for tissue-engineered scaffolds and drug delivery systems [71]. GelMA, specifically, can be electrospun and photo-crosslinked to form HFs. These materials promote stem cell migration, control their differentiation, inhibit glial scar formation, and support angiogenesis [51,72]. In the realm of synthetic polymers, they have been confirmed to exhibit reproducibility and can be tailored with outstanding biocompatibility to mitigate immune rejection responses [63,73]. They can also be designed to present abundant cross-linking sites to advance HF formation and offer exceptional mechanical properties to support tissue regeneration [74,75]. For example, HFs composed of CS-graft-poly (N-isopropylacrylamide) and PEO were engineered for the containment of BSA, with a release rate responsive to alterations in ambient pH and temperature. This innovation advances the development of stimuli-responsive biomaterials in the fields of drug delivery and tissue engineering [76]. Nevertheless, synthetic polymer-based HFs often lack molecular recognition sites, which impede cell/tissue communication pathways and consequently reduce therapeutic efficacy [77]. In this context, the functional HFs should be exploited by fully harnessing the distinctive benefits of natural materials and synthetic polymers, thereby developing a high-performance therapy targeting specific biological processes for tissue regeneration.

Overall, the effective production of HFs necessitates materials with plentiful hydrophilic groups on the main/side chain of molecules for cross-linked network formation. It also requires the materials to exhibit superior solubility in an appropriate solvent and enable molecular entanglement to facilitate fiber generation. Hence, long-chain polymers possess a greater propensity for HF fabrication compared to low molecular weight species; linear polymer topologies promote more effective molecular entanglement than branched analogues. Compared to synthetic polymers, natural polymers exhibit a favorable molecular composition. Still, they are limited in chemical manipulability that can be tailored to enhance the physical attributes (e.g., mechanical properties and degradability) of HFs. Besides, the diversity in monomer synthesis and polymerization techniques allows for the creation of innovative biomaterials that meet precise criteria [92]. Notably, there is a growing recognition within the scientific community: the characteristics of synthetic polymer chemistry complement those of natural materials, and vice versa. This acknowledgment has spurred the advancement of hybrid polymers for the fabrication of multifunctional HFs [93].

### 3.2 Preparation strategies for HFs

Since the preparation process of HFs involves hydrogel formation and fiber generation, their preparation methods can be divided into three categories in theory: 1) fiber generation followed by hydrogel formation; 2) simultaneous occurrence of fiber generation and hydrogel formation; 3) hydrogel formation followed by fiber generation. Due to the challenges in integrating hydrogels into micro-/nanofibrous architectures, current strategies primarily concentrate on two approaches: generating fibers followed by hydrogel formation, and simultaneously generating both fibers and hydrogel. For clarity, this section focuses on the recently developed strategies for HFs based on the preparation methods of micro-/nanofibers.

**Fig. 4:**
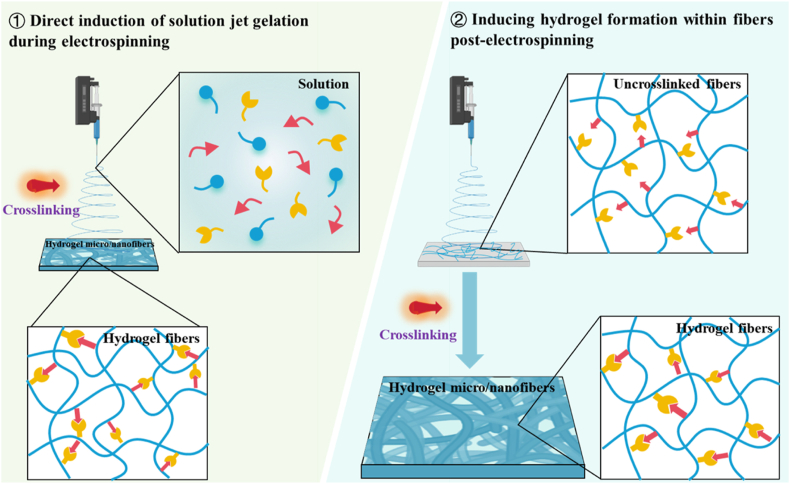
Electrospinning-based strategies for HFs generation, including simultaneous occurrence of fiber generation and hydrogel formation by promoting fiber-fiber crosslinking during fiber spinning (left) and hydrogel formation followed by fiber generation, by promoting fiber-fiber crosslinking after fiber spinning (right).

Of all the current strategies available for synthesizing micro-/nanofibers, electrospinning ranks as one of the most established and widely adopted techniques [15]. As such, electrospinning-based approaches have been extensively employed for the fabrication of HFs [24]. Up to date, the developed electrospinning-based strategies could be categorized into two types: (1) direct control of hydrogel formation during electrospinning and (2) promotion of HF formation after electrospinning (Fig. 4). Specifically, the strategy involving the direct control of hydrogel formation during electrospinning entails the occurrence of molecular crosslinking within the solution jet before micro-/nanofiber formation. As an example, Wei et al. employed a UV light source positioned at the outlet of the spinning solution. Upon UV light exposure, the dynamically extruded spinning solution monomer underwent free radical polymerization, leading to the crosslinking of the polymer chains and subsequent formation of HFs after immersion in a pre-prepared water-soluble thermal initiator solution [94]. Another approach, which promotes the generation of HFs after electrospinning, involves inducing crosslinking of polymer molecules within the fiber framework. This method entails the formation of micro-/nanofibers through electrospinning, followed by the initiation of hydrogel formation through crosslinking techniques such as photopolymerization or metal-chelation. For instance, GelMA was dissolved in hexafluoroisopropanol, and an arrayed GelMA micro-/nanofiber scaffold was fabricated *via* electrospinning. Following immersion in a photocrosslinking solution and exposure to UV light for 60 min, HFs were successfully formed [84]. In the examination of these methods, it is readily apparent that the fundamental difference resides in the timing of stimulus application for hydrogel induction. The synchronized spinning and hydrogel formation approach minimizes time expenditures. In contrast, the alternative method, which necessitates post-fiber-formation crosslinking, presents advantages in terms of reduced facility requirements and enhanced cost-effectiveness. Indeed, the properties of HFs can be tailored by adjusting parameters such as fibrous structure, fiber stiffness, and crosslinking density.

**Fig. 5:**
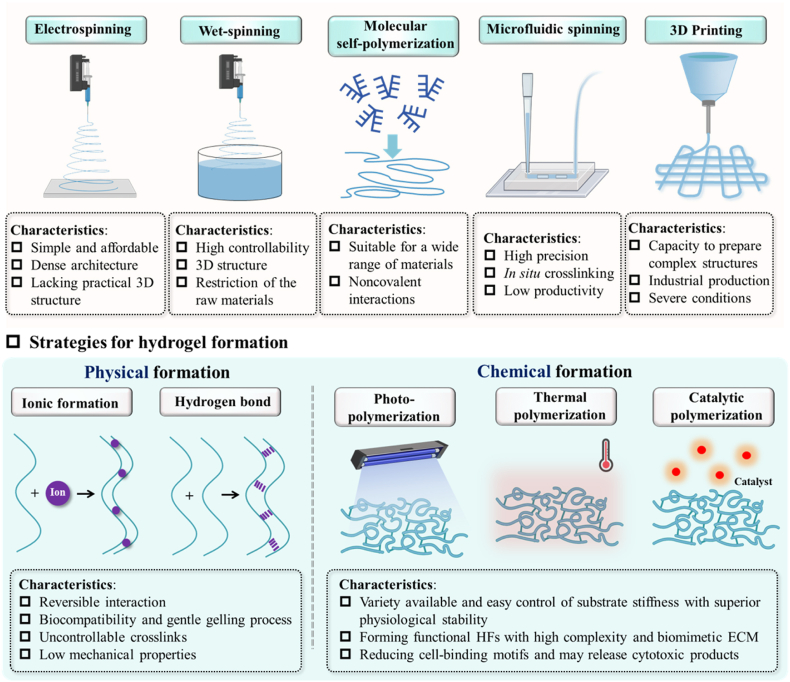
Strategies and characteristics of micro-/nanofiber fabrication and hydrogel formation for HF preparation.

Furthermore, in view of the generation principle of HFs, if a biomaterial possesses hydrogel-forming potential and can be processed into micro-/nanofibers, it holds promise for the development of HFs. For instance, to date, various strategies beyond electrospinning have been exploited for the generation of micro-/nanofibers, spanning established methods (e.g., self-assembly, polymerization, and template-based synthesis) and emerging approaches (e.g., solution blow spinning, microfluidic spinning, centrifugal jet spinning, and 3D printing) [95]. Meanwhile, strategies for inducing molecular crosslinking among polymeric chains to form hydrogels encompass physical cross-linking mechanisms (e.g., ionic cross-linking, hydrogen bond formation, and electrostatic interaction) and chemical cross-linking pathways (e.g., addition polymerization, condensation polymerization, and enzyme-catalyzed reaction) (Fig. 5) [45]. In this context, based on the inference drawn, the presence of a polymer with crosslinking sites in the fiber-preparation material, or a polymer that can be functionally modified to yield fibers with crosslinking sites, enables the development of novel HFs through the strategic selection of hydrogel and micro-/nanofiber fabrication techniques. For example, inspired by spider silks, Wu et al. designed a hydrogel fiber featuring ionic and crystalline domain crosslinking by leveraging the ionic coordination and Hofmeister effects of inorganic salts after the implementation of an improved self-lubricating spinning strategy [96]. Yang et al. synthesized a hierarchical fibrous hydrogel exhibiting high alignment and remarkable flaw insensitivity. This was accomplished by engineering a wet rotary jet spinning platform, subsequently followed by a salting-out process [97].

Notably, each strategy for hydrogel formation and micro-/nanofiber generation exhibits distinct advantages and limitations. Electrospinning, for example, is a straightforward and cost-effective method for producing nanofibers [98]. However, conventional electrospun HFs predominantly consist of densely packed fiber layers. Their small pore sizes, low porosity, and lack of practical 3D structures limit cell ingrowth [99]. Wet-spinning involves the coagulation of hydrogel precursor solution in a bath, followed by poor-solvent-induced phase separation and subsequent crosslinking. This approach is a predominant method for mass-producing fibers due to its technical maturity and high degree of controllability [97]. The resulting HFs can be configured into 3D structures through braiding, weaving, or embroidering, and their cross-sectional shapes can be tailored by altering the extruders. However, a solidification process is essential for wet-spinning, thus restricting the materials that can be used for hydrogels.

Additionally, there are challenges in producing HFs with diverse structures, controllable compositions, and organized functions [100]. Molecular self-polymerization involves the spontaneous formation of well-organized structures *via* noncovalent interactions [25]. It applies to a wide range of native materials for constructing HFs, including proteins, polysaccharides, amino acids, and peptides. Due to their physical and chemical similarities to ECM, these self-assembled HFs show great promise in tissue repair [101]. However, self-assembly results from noncovalent interactions, leading to poor mechanical properties of the HFs. Therefore, additional crosslinking or the introduction of a secondary network is necessary. Microfluidics involves precision-machined microchannels that inject two or more immiscible liquids with coaxial flow into a customized microfluidic chip to continuously produce fiber-shaped streams, which are cross-linked *in situ* [102]. HFs with controlled sizes and structures can be made by adjusting the concentration and viscosity of precursor solutions, as well as the channel geometries of the microfluidic chips.

With the ability to incorporate living components (e.g., cells, proteins, and DNA), microfluidic spinning has garnered significant interest for HF-based therapeutic applications in damaged tissues [103]. However, achieving a high yield of HFs remains a considerable challenge for this method. 3D printing is an accessible method for preparing HFs with complex structures and enabling their industrial production [20]. It involves extruding a hydrogel precursor through a printing nozzle and solidifying it. During continuous extrusion, viscoelastic behaviors of the hydrogel precursors are required to ensure good shape fidelity and precise size control of HFs, while *in situ* fiber solidification is necessary to ensure the continuous fabrication of HFs with good shape fidelity.

Regarding hydrogel formation, the types of crosslinking applied for HF formation include physical crosslinking (e.g., ionic crosslinking and hydrogen bond) and chemical crosslinking (e.g., photo-polymerization, thermal polymerization, and catalytic polymerization). Physical crosslinking is known as a reversible interaction [45]. Compared to chemical crosslinking, HFs formed using gentle physical crosslinking exhibit significantly increased biocompatibility, making them suitable for tissue regeneration [23]. However, their stiffness is difficult to control precisely due to uncontrollable crosslinking and the inevitable influence of surrounding environments, such as pH, ion concentration, and temperature. Additionally, they suffer from other limitations, such as inadequate mechanical properties, restricting their applications in stiff tissue repair. Chemical (i.e., permanent) crosslinking offers a variety of substrate stiffness options with easy control and superior physiological stability [45]. It can be used to develop functional HFs with higher degrees of complexity and biomimetic ECM, of an interpenetrating network within single fibers of HFs. However, the employment of chemical crosslinking may consume the reactive groups of biomolecules, reducing the availability of essential cell-binding motifs and potentially releasing cytotoxic products once metabolized.

## 4 Research progress of HFs for soft tissue repair and regeneration

Cell-matrix interactions, involving cell adhesion, migration, growth, and ECM deposition, play a pivotal role in tissue remodeling and regeneration [104]. Hydrogels enable enhanced cell metabolism and improved matrix remodeling, whereas fibrous networks facilitate deeper cellular infiltration and superior cell contractility. Consequently, the integration of fibrous structures into hydrogel networks has been demonstrated to yield superior biological functions and provide a desirable environment for cell-matrix interactions. For example, fibrous structures of HFs could provide structural support and contribute tensile strength to connective tissues subjected to tension. Their high water ensures adequate lubrication and enhances compression resistance in biological tissues during movement, while also facilitating the diffusion of bioactive substances.

Furthermore, accumulating evidence demonstrates that a mismatch in mechanical properties between implanted biomaterials and native tissue might cause adverse health effects, ranging from micro-injuries and cell damage to inflammation, fibrosis, and necrosis [105,106]. HFs address this issue by mimicking the ECM—a composite of fibrous networks within a hydrogel matrix that provides the unique mechanical properties and adaptability of biological soft tissues. This ECM-mimicking characteristic underpins the strong mechanical biocompatibility of HFs, making them widely explored candidates for soft tissue repair applications (Table 2). This section primarily presents the research progress on HFs in the regeneration of damaged soft tissue, including nerve, skin, cardiovascular system, and skeletal muscle.

**Table 2:** Research progress of HFs in tissue repair and regeneration.

Area	Hydrogel fibers	Fabrication Method	Results	Ref.
Nerve	Aligned fibrin/self-assembled peptide interpenetrating HFs	Electrospinning and molecular self-assembly	Promoting the recovery of motor function; Promoting nerve-related gene expression and activating PI3K/Akt and MAPK pathways.	[116]
	RGI/QK peptide grafted directional CS HFs	Electrospinning and molecular self-assembly	Orienting the schwann cells and promoting their proliferation and neurotrophic factors secretion; Enhancing vascular penetration and promoting nerve regeneration.	[65]
	Self-assembled peptide HFs	Electrospinning and molecular self-assembly	Promoting cell adhesion, proliferation and lineage differentiation of NSCs.	[114]
	CNT/GelMA HFs	Electrospinning and photocrosslinking	Mimicking the aligned structure, conductivity, and softness of the neural axons to support PC12 cells viability and induce NSCs neuronal differentiation.	[162]
Skin	Electrospinned GelMA/poly (L-lactic acid) HFs	Electrospinning and photocrosslinking	Promoting the attachment, growth, and proliferation of human dermal fibroblasts.	[163]
	Catechol bound alginate HFs	Wet spinning and CaCl_2_ crosslinking	Maintaining the shape fidelity of the 3D structure and encapsulating cell density during the cultivation process.	[70]
	GelMA HFs	Electrospinning and photocrosslinking	Supporting cell adhesion, proliferation, and allowing cells to infiltrate the 3D scaffold for skin repair and regeneration.	[121]
	DCMC crosslinked gelatin PEG composite HFs	Stretch spinning and DCMC protein crosslinking	Reducing the swelling degree of HFs and exhibiting a 3D porous network for wound exudates absorption and a moist environment maintenance.	[164]
Cardiovascular system	Alginate/gelatin HFs	Wet electrospinning and photo crosslinking	Supporting the proliferation of MSCs, and maturing human iPSC-derived ventricular myocytes.	[79]
	Pectin HFs	Electrospinning and ADH crosslinking	Promoting MSC differentiation into vascular SMCs and ECs.	[67]
Skeletal muscle	Slotted solid and hollow HFs	Wet spinning and CaCl_2_ crosslinking	Exhibiting controlled myogenic differentiation and morphological changes induced in C2C12 myoblasts.	[154]
	Fibrin HFs	Electrospinning and CaCl_2_/thrombin crosslinking	Increased number of myosin heavy chain positive cells in regenerated muscle markers such as embryonic myosin and mature muscle markers.	[152]
	Slender GelMA HFs	Stretch spinning and photo-crosslinking	Enhancing the metabolic activity of cell-inoculated fibers and promoting the production of structures closing to natural mammalian muscle tissue.	[156]

### 4.1 Nerve regeneration

HFs that mimic the nerve microenvironment enhance the regeneration process by guiding nerve cell migration and promoting axon extension.

Within the healthy spinal cord, ECM forms an intricate 3D network with intrinsic matrix stiffness spanning 0.5–50 kPa [107]. It provides anchorage and support for neuronal and non-neuronal cells. Following SCI, ECM breakdown exacerbates nervous tissue destruction and disrupts bidirectional communication of basic neurological functions between the brain and spinal cord. Consequently, optimizing the ECM milieu for repair represents a viable therapeutic strategy. Accumulating evidence has demonstrated that neural tissue repair necessitates materials with high water content to mimic the natural ECM environment [108]. Mechanically, these materials should match the stiffness of surrounding soft tissues to prevent stress shielding while retaining elasticity to accommodate dynamic nerve stretching [11]. Structurally, anisotropic fiber alignment or microchannels are required to guide directional axonal regeneration, alongside porous or hollow fiber architectures to facilitate vascularization and metabolite exchange [109]. Bioactive signals, such as neurotrophic factors, should be incorporated to promote neuronal survival and axonal growth, whereas anti-inflammatory factors can suppress scar formation. Additionally, electrical conductivity is essential to synergize with electrical stimulation in enhancing neuronal differentiation and migration [110].

**Fig. 6:**
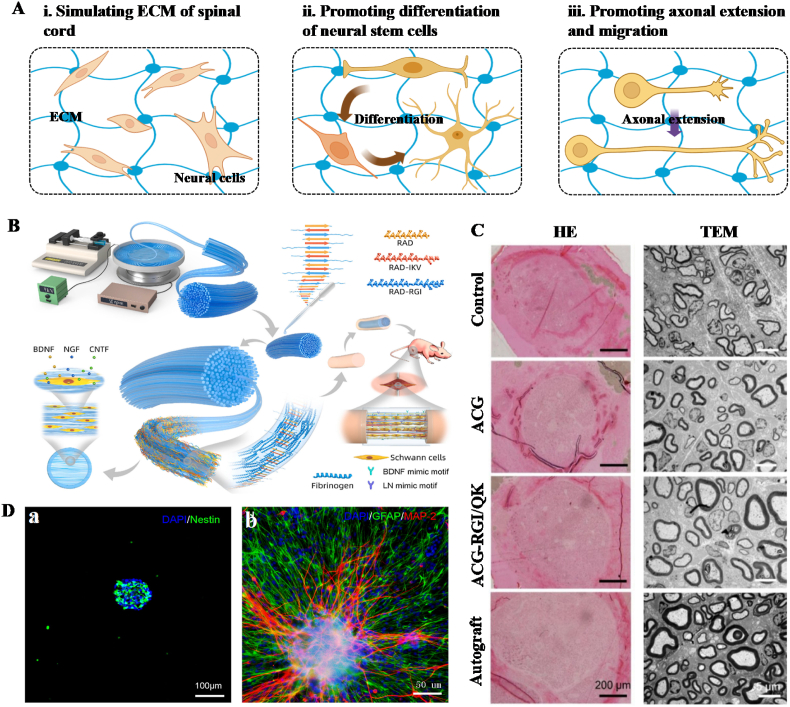
Effects of HFs on nerve regeneration: (A) Schematic diagram of the merits of HFs for nerve regeneration; (B) Schematic diagram of the fabrication of AFG/fSAP interpenetrating hydrogel fibers for nerve regeneration [116]; (C) Modification of CS HFs with RGI peptides and QK peptides to regenerate nerve fibers [65]; (D) FGLmx-functionalized peptide HFs stimulated differentiation proportion of neuronal (MAP-2 positive, red) and astrocytic (GFAP positive, green) cells [114]. Copyright was granted by Elsevier (License Number: 5853630534658).

Attributing to their softness that closely resembles that of the spinal cord ECM, HFs have emerged as promising biomaterials for repairing the damaged nervous system [11]. This feature facilitates the directional differentiation of NSCs into neuronal cells. Their high viscoelasticity not only provides a conducive environment for neuronal cell proliferation but also allows the substrate to withstand displacement at implantation sites during deformation without requiring spinal canal protection [86,111,112]. Meanwhile, the 3D fibrous structure of HFs mimics the anatomical arrangement and directional orientation of nerve fibers. This provides guidance and cues for axon growth and functional recovery, thereby establishing a pro-regenerative environment (Fig. 6A) [[88], [89], [90]]. Considering that NSCs transplantation holds promise for treating SCI, HFs provide an ideal microenvironment for culturing NSCs *in vitro*. They can significantly enhance the survival, proliferation, and differentiation of NSCs into neurons and oligodendrocytes, while promoting neurite outgrowth and remyelination at injury sites to facilitate neural recovery in SCI [113,114]. To enhance the nerve guidance capabilities of HFs, the integration of bioactive molecules (e.g., drugs, growth factors, or small molecules) has been established as an efficacious strategy. Growth factors possess the capacity to direct axon elongation, sustain axon viability, and encourage axon regeneration [90,91]. As such, a variety of growth factors or growth factor-derived peptides (e.g., BDNF [112] and NGF [113], and VEGF [65]) have been incorporated into HFs to facilitate nerve regeneration (Fig. 6B). An example of this involves the utilization of a CS HF system loaded with a combination of RGI peptides and QK peptides, which mimic BDNF and VEGF, respectively. These micro-/nanofibers were used as a nerve conduit filler to address sciatic nerve defects in rats, resulting in notable enhancements in nerve cell function, nerve regeneration, and vascular infiltration during the initial phase of injury [65] (Fig. 6C). Another strategy focused on the development of FGLmx-functionalized peptide HFs incorporating the FGL motif derived from the NCAM. This motif stimulates NSC differentiation (Fig. 6D), synaptogenesis, and memory consolidation, leading to the successful repair of injured spinal cords [114]. Additionally, alginate HFs have been utilized for the encapsulation of neuroprotective drugs such as RC-33, demonstrating a notable ability to confer substantial neuroprotection and facilitate nerve regeneration in the treatment of spinal cord injuries [115].

Overall, HFs possess the capability to replicate the microenvironment of neural tissue and support the migration of neuronal cells and axon extension. This renders them promising materials for neural regeneration and repair. Accumulating evidence has confirmed that cell behavior modulated by HFs may be influenced by cell-cell interactions, such as those between neurons and glial cells [88,112]. Nevertheless, the specific cellular responses and molecular mechanisms orchestrated by HFs in promoting nerve regeneration remain not fully elucidated, warranting further exploration. Moreover, despite the demonstrated potential of HFs in promoting nerve regeneration and repair, they have not yet achieved comprehensive success in restoring nerve functions. Consequently, to target the mechanism underlying functional recovery, optimizing the pertinent properties of HFs to expedite neuron integration during nerve repair is essential.

### 4.2 Skin healing

HFs create a highly absorbent microenvironment to facilitate the absorption of excess exudates, promote efficient hemostasis, and prevent wound contracture and contour deformation, contributing to the healing process.

**Fig. 7:**
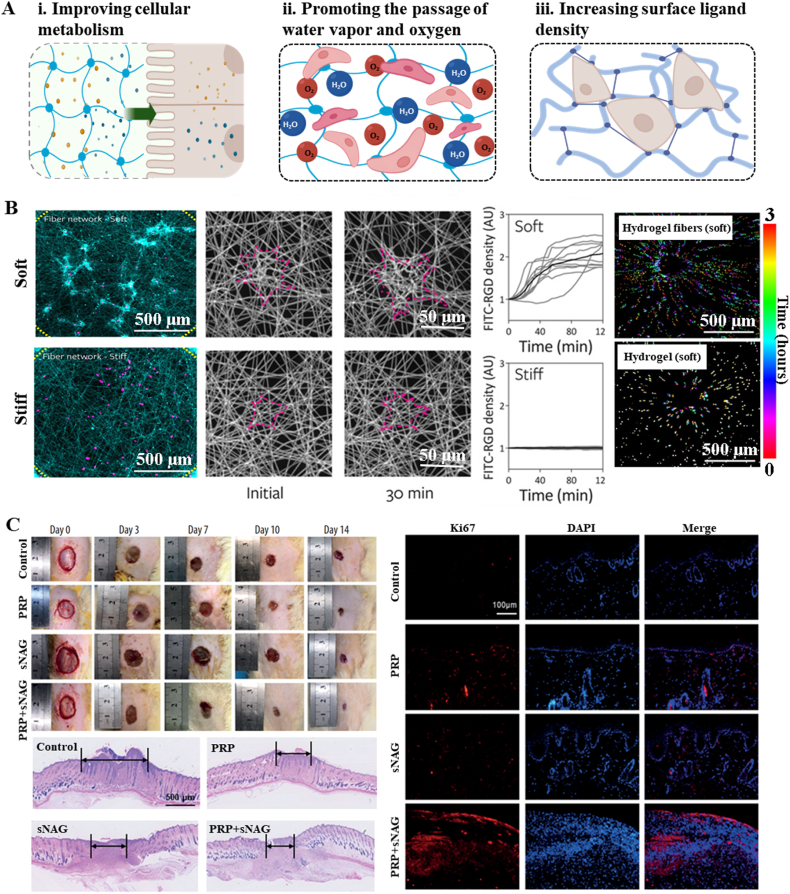
Effects of HFs on skin healing: (A) Schematic diagram of the merits of HFs for skin healing; (B) Low stiffness of HFs enabled cell-mediated reorganization of the material and clustering of adhesive ligands local to the cell. Dotted lines indicate the periphery of the suspended network [122]; (C) Incorporation of HFs with PRP to accelerate wound healing [130]. Copyright was granted by Elsevier (License Number: 5994741189869).

The degree of wound healing primarily depends on cell-matrix interactions, and rapid healing of the wound surface in tissue engineering is promoted mainly by providing a suitable substrate environment for fibroblast proliferation. Skin ECM exhibits a complicated fibrous structure with a macroscopic elastic modulus of 100–200 kPa (in contrast to the microscale moduli of 0.1–10 kPa), composed of collagen, elastin, laminin, polysaccharides, and proteoglycans [117], and possesses a high water-absorbing capacity. Therefore, skin substitutes should mimic the tissue structure and function of skin ECM to the greatest extent to improve physiological function [21,118,119]. Physical parameters of scaffolds influence cellular behavior and tissue regeneration due to their ability to modulate biological processes and determine cell fate, similar to the biochemical signals [45]. Hence, HFs have demonstrated significant efficacy in enhancing skin repair, attributable to their high hydration capacity, favorable elasticity, stimuli-responsiveness, high specific surface area, ease of processing into woven structures, and robust mechanical properties (Fig. 7A). They provide a suitable substrate environment for cell proliferation and immersion, and mechanical compatibility for wound healing treatment [24,120]. For example, their excellent hydrophilic properties allow HFs to maintain moisture balance on the surface of skin trauma [118], and the fibers maintain mechanical compliance and elastic stability of scaffolds despite high water absorption on skin, which is often subject to high tension. This biomimetic size and shape, resembling natural ECM protein fibrils, not only maximizes cell-matrix interaction [121,122] but also facilitates rapid hemostasis [121]. The prompt hemostasis capabilities may be attributable to the reduction of complications such as contracture and contour deformation commonly associated with conventional wound healing approaches [120]. As cells tend to show increased migration in flexible matrices compared to rigid scaffolds [123], the presence of low-stiffness fibers in HFs enables active cells to recruit neighboring fibers, resulting in enhanced ligand density on the cell surface (Fig. 7B). This process, in turn, stimulates cell adhesion and associated signaling pathways, ultimately facilitating cell migration [122]. Certainly, HFs can match the mechanical properties of human skin by carefully selecting appropriate raw materials. As soft biomaterials, these wound dressings are easily and painlessly removed by gentle swabbing during frequent dressing changes [124].

By selecting different materials, HFs with a composition similar to that of natural ECM can be prepared. HA is a prominent constituent of the skin ECM, actively secreted by fibroblasts and keratinocytes continuously [125]. In preparing HA-based HFs, Ji et al. employed PEO, a high-molecular-weight polymer, as a viscosity modifier to assist in forming fibers from the HA aqueous solution [126]. To enhance the bioactivity of scaffolds for chronic refractory wound, HFs could be utilized as vehicles for localized release of therapeutic agents, including various therapeutic compounds or molecules (e.g., antibacterial agents [127], metal ions [24], and growth factors [128]), for the prevention of wound infections, acceleration of the wound healing process, and reduction of scar formation [24] (Fig. 7C). For example, self-assembled peptide HFs were functionalized with N-acetylglucosamine to enhance antibacterial properties and stimulate angiogenesis of the biomaterials, designed explicitly for chronic ulcer wounds [129]. Moreover, Zhang et al. formulated a poly (*N*-acetaminophen) micro-/nanofiber hydrogel incorporated with PRP, which demonstrated notable effects in promoting fibroblast proliferation, suppressing apoptosis, facilitating new blood vessel formation, and alleviating superficial neuropathic scar pain [130]. Given the critical role of surface chemistry in matrix-guided macrophage polarization, the transition of monocytes to an M2-like macrophage phenotype was achieved through phosphorylation of plant-derived nanofibrillar cellulose, and further demonstrated that cells within these nanofibrillar cellulose hydrogels exhibit a distinct cytokine profile compared to those cultured in two dimensions, secreting 2.6-fold higher levels of interleukin-1β and 1.2-fold greater amounts of interleukin-10 [131]. Capitalizing on the potential of structural anisotropy to orchestrate multicellular regulation through physical contact and cellular mechanosensing pathways, Kim et al. developed a highly oriented anisotropic nanofiber hydrogel to induce shape-driven polarization of macrophages, which was further functionalized with a VEGF-mimetic peptide [132]. The combined system enhanced crosstalk among immune regulation, neurogenesis, and tissue regeneration, ultimately accelerating diabetic wound healing through a triadic synergy of immune-angiogenic-neurogenic microenvironments. To prevent and treat wound infections, Wang et al. prepared a pH-switchable antimicrobial HFs *via* a self-assembly strategy that exhibited acidic pH-responsive release profiles [133]. Sutures are typically employed to close wounds, reduce inflammation, and alleviate pain. Guan et al. developed a lotus-like bacterial cellulose hydrogel fiber for surgical sutures that possess an elastic modulus comparable to that of human skin [134]. The bionic spiral structure enables synchronous deformation with human tissue and effectively avoids secondary injuries caused by incisional wounds.

Despite notable progress, challenges persist in the implementation of HFs for skin repair. These challenges encompass issues such as the inadequate purity of biomaterials sourced from the natural ECM, as well as the necessity for enhanced control over drug release [135]. While HFs have exhibited significant therapeutic advantages in wound healing [24], additional investigation is required to elucidate the physiological mechanisms and underlying molecular pathways associated with material-induced skin regeneration. Moreover, the mechanical characteristics and toughness of current HFs differ markedly from those of human skin. The absence of critical elements like blood vessels, hair follicles, and sweat glands limits the effectiveness of HFs in promoting skin restoration and hinders the degradation of injured skin [24]. Mitigating these constraints necessitates the development of multifunctional HFs that replicate the intricate structure and functionality of natural skin.

### 4.3 Cardiovascular repair

*HFs offer biomimetic properties and viscoelasticity, enabling them to adapt to cardiac motion, coordinate the inflammatory response, and facilitate the development of microvascular networks for cardiovascular repair*.

Cardiac muscle contains a hierarchically organized fibrous network with stiffness values ranging from 4 kPa to 320 kPa [136], comprising epimysial, perimysial, and endomysial fibers. Epimysial fibers encase the heart muscle and prevent excessive stretching, while perimysial fibers surround cardiac muscle bundles and form a fibrous meshwork to connect bundles. Endomysial fibers interact with individual cardiomyocytes and penetrate their cell membrane to engage with cytoskeletal proteins. These fibrous structures contribute to excellent mechanical properties, guide cell maturation, and enable electrical and mechanical coupling between cardiac cells. Besides, the 3D anisotropic fibrous architecture ensures circular contraction, yielding robust pump function. In this context, mechanically, biomaterials for cardiovascular repair should exhibit sufficient elasticity to withstand myocardial contraction stress [137]. Structurally, anisotropic alignment should be performed to mimic myocardial fibers, and pre-vascularized networks are required to prevent post-transplant ischemic necrosis. Electrically, conductivity is essential to ensure synchronous electrical signal transmission and avoid arrhythmia.

**Fig. 8:**
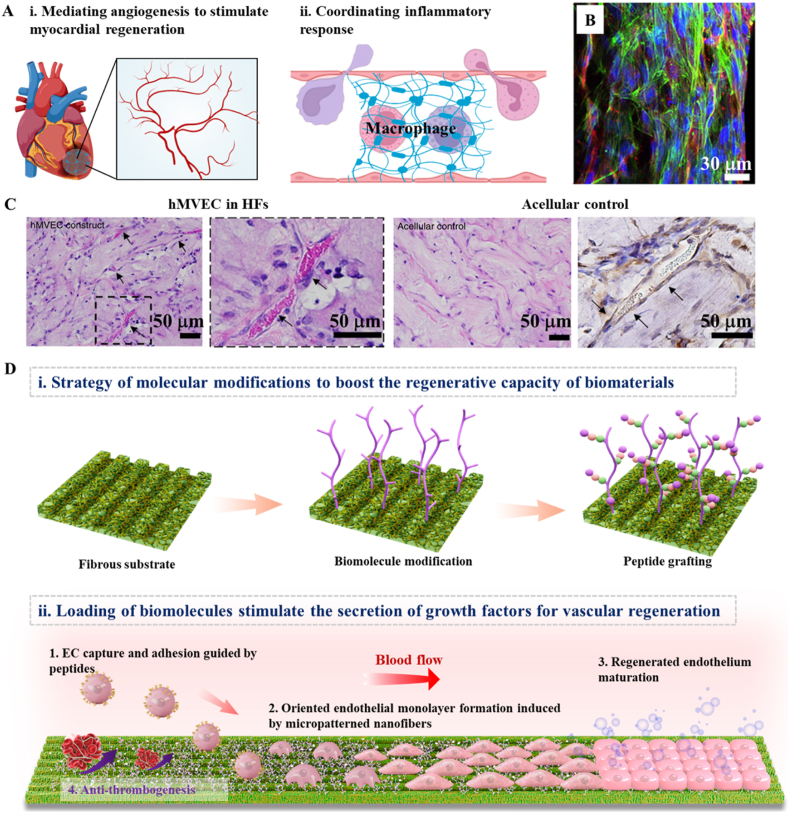
Effects of HFs on cardiovascular repair: (A) Schematic diagram of the merits of HFs for cardiovascular repair; (B) PAN-based HFs enabled the tailoring of the biological traits of supramolecular structures to achieve specific cell behavior and tissue regeneration [144]; (C) Implantation of hMVEC promoted vessel formation within the HFs construct [140]; (D) Step-wise modification of biomolecules on vascular fibrous grafts for programmed endothelial healing [145]. Copyright was granted by Elsevier (License Numbers: 5853631065115 and 5884090411742).

HFs, such as self-assembled peptides [138], alginate salts [139], pectins [67], and polyelectrolytes [140], have garnered increasing attention as scaffolds for cardiac repair due to their biomimetic viscoelasticity, physicochemical versatility, and self-adaptability. Initially, HFs were utilized to enhance myocardial cell cultivation, enabling the transition from 2D to 3D *in vitro* cultures. For example, Ikonen et al. demonstrated the capability of hydrophilic self-assembled HFs to support the cultivation of neonatal rat CMs and human embryonic stem cell-derived CMs [141], indicating their potential in expediting heart tissue repair. To facilitate 3D stem cell culture and myocardial cell maturation, Seidelin et al. introduced an innovative approach for fabricating 3D macroporous alginate/gelatin HFs with enhanced cell adhesion, migration, and maturation [47]. This study establishes the groundwork for the systematic design and advancement of 3D cell culture scaffolds, fostering cell maturation and cardiovascular repair. Recently, HFs have been confirmed to improve cardiac function in cardiovascular disease patients by mimicking the ECM and orchestrating the inflammatory response in the healed heart. For example, HFs enhance fibroblast transcription of inflammatory pathways *via* FGF-2 and toll-like receptor 9-dependent signaling, resulting in the secretion of angiogenic factors such as VEGF and inflammatory cytokines [142]. This process promotes microvascular network formation and myocardial regeneration [128,129,143] (Fig. 8A). Since heart disease increases myocardial viscoelasticity that impedes diastolic filling and decreases cardiac output [133,134], the high viscoelastic properties enable HFs to adapt to cardiac motion without displacement or detachment. HFs also allow customization of supramolecular structure, biological traits to achieve specific cell behaviors and tissue regeneration [144](Fig. 8B). Aligned endothelial vessel formation is an effective pre-vascularization strategy for promoting cardiovascular repair. Leong et al. fabricated aligned and spatially defined pre-vascular tissue constructs containing endothelial vessels by assembling individually customized polyelectrolyte HFs with hMVECs. Within 24 h, these constructs formed stable, neatly arranged endothelial blood vessels *in vitro* (Fig. 8C), which anastomosed with the host circulation in a mouse model [140]. Molecular modifications are considered an effective strategy to enhance the regenerative capacity of HFs for ischemic myocardial capillary networks and the restoration of blood flow (Fig. 8D–i) [145]. Direct approaches involve the incorporation of growth factors (e.g., VEGF and FGF) and PLs [146], while indirect approaches load bioactive substances to stimulate growth factor secretion, thus facilitating vascular growth and augmenting regenerative potential (Fig. 8D–ii) [145]. For vascular system repair, the physicochemical and physiological similarities between HFs and natural tissues create favorable environments for EC and SMC proliferation and ECM secretion, contributing to vascular development [60,129].

Nevertheless, several challenges still need to be addressed to achieve comprehensive tissue repair and functional rehabilitation in the cardiovascular system. One challenge lies in the mediation of angiogenesis, as the dense internal fiber structure of HFs hinders the formation of perfusion and vascular networks within the scaffold. Besides, the involvement of HFs in myocardial fibrosis and immune-associated cardiovascular diseases remains largely unexplored [143]. Moreover, studies have indicated that functional HFs could be engineered by combining mechanical strength with biochemical cues to induce the differentiation of MSCs into ECs [147]. Nevertheless, under high-intensity culture conditions, HFs have been shown to drive MSC differentiation towards SMCs, leading to increased cellular contractility and traction [148]. These observations highlight the challenge of balancing the mechanical strength of HFs to fulfill the diverse requirements of cardiovascular regeneration. Therefore, it is essential not only to pursue a biomimetic stiffness resembling mature tissues but also to consider the impact of scaffold mechanics on cardiovascular repair, regeneration, and developmental processes to attain optimal repair outcomes [67].

### 4.4 Skeletal muscle repair

HFs display adjustable terrain and biochemical signal stimulation that synergistically induce cell elongation and infiltration, thereby facilitating myotube formation and myoblast differentiation.

**Fig. 9:**
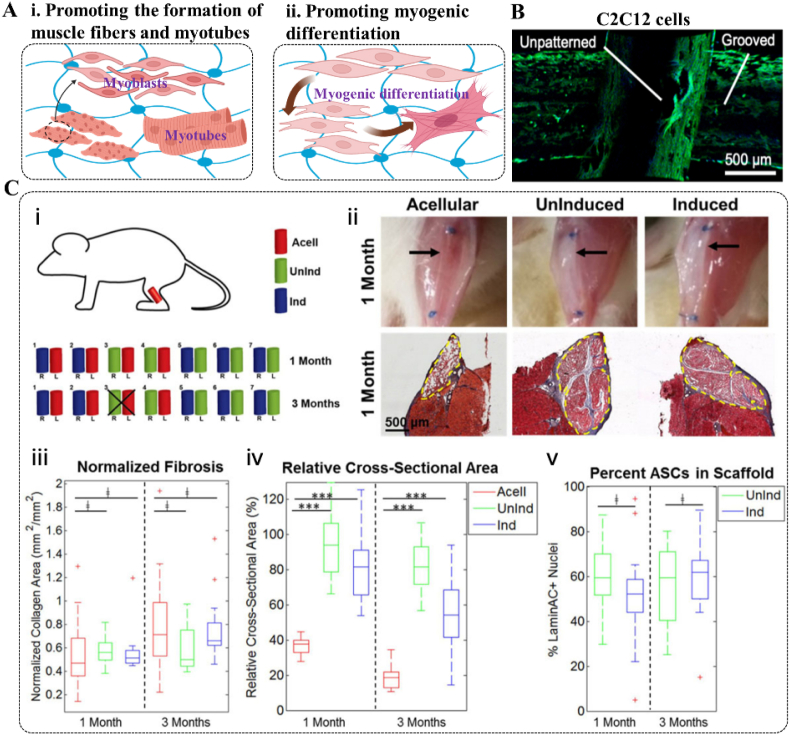
Effects of HFs on skeletal muscle repair: (A) Merits of HFs for promoting the formation of muscle fibers and myotubes (i) and enhancing myogenic differentiation (ii); (B) The groove micro-/nanofibers enabled control over morphological changes in C2C12 myoblasts [154]; (C) Pre-seeding of ASC on hydrogel fibers promoted myosin mature [152]: (i) HFs were implanted per mouse and harvested at 1 or 3 months; (ii) differences between acellular and cell-seeded groups immediately before harvest. Black arrows illustrate fiber location within the defect site, and dashed yellow lines denote fiber boundaries; (iii) no significant differences appeared in the quantification of normalized fibrosis; (iv) cell-seeded samples had significantly higher retention of cross-sectional area than acellular samples; (v) no significant difference appeared in the quantification of LaminAC + nuclei percent. Copyright was granted by Elsevier (License Number: 5884090794541).

Skeletal muscle (matrix stiffness ∼20 kPa [149]) comprises muscle fibers, connective tissue (endomysium, perimysium, epimysium, and tendons), blood vessels, and neural networks. Among these components, skeletal muscles and tendons display distinct highly anisotropy in cell organization, which facilitates effective force transmission and contraction [150]. The connective tissue provides a supportive framework for skeletal muscles and enables synchronized muscle fiber contraction during exercise. Tendons, known for their dense fiber structure, possess high tensile strength and serve as connections between muscles and bones, as well as between bones themselves. In this context, the scaffold used to support skeletal muscle regeneration has been designed to provide appropriate terrain stimulation that promotes the formation of densely packed and highly organized muscle fibers [151,152]. Topology-induced stimulation encouraged muscle progenitor cells (myoblasts) to form a biomimetic structure resembling parallel lines found in natural skeletal muscle, thus enhancing the formation of myotubes (Fig. 9A). To further mimic the arrangement of collagen fibrils, collagen hydrogel microfibers with directional and parallel alignment have been developed [153], which not only provide typical topographic stimuli but also supply biochemical cues that synergistically promote myotube formation and myoblast differentiation. Since the weaving morphology and porosity of HFs were verified to influence myogenic differentiation and morphological changes, three-dimensional arrayed electrospun HFs with either weaving or stacking morphology have been fabricated [154]. These stacked scaffolds not only exhibit superior cell infiltration compared to woven scaffolds, resulting in higher total cell count and ECM content, but also possess good mechanical properties that promote the expression of tendon-specific markers, thereby facilitating tendon generation [117]. Furthermore, to improve the morphology of single fibers, Mirani et al. investigated a novel biomanufacturing method to produce grooved solid and hollow HFs [154]. These groove micro-/nanofibers markedly induced the alignment of myoblasts and enable control over myogenic differentiation and morphological changes in C2C12 myoblast (Fig. 9B), depending on the size of the grooves.

Electrical and mechanical stimulation can promote the differentiation and maturation of muscle cells. Hence, enhancing the conductivity of HFs promoted the differentiation of muscle stem cells, known as satellite cells, which express numerous gene markers specific to skeletal muscle tissue [155]. Besides, the biological properties of HFs could be enhanced by combining them with stem cell therapy or incorporating bioactive molecules. For example, Jordana et al. seeded human ASCs onto fibrin HFs and implanted the cell-HF constructs into a robust mouse VML deficiency model. Results confirm that while muscle regeneration was not observed *in vitro*, ASCs combined with fibrin HFs moderately enhanced muscle reconstruction *in vivo* compared to acellular substrates following severe trauma [152] (Fig. 9C). Additionally, the incorporation of plant-based virus nanoparticles into HFs noticeably increased cellular metabolic activity, improved cell adhesion and alignment, and promoted the formation of muscle tissue structure [156].

The applications of HFs in skeletal muscle repair have been comparatively limited compared to their application in accelerating the repair of other tissues, primarily due to constraints on mechanical properties [157]. Moreover, the advancement of engineered constructs using HFs still lags behind the intricacy and maturity of natural muscles [158]. Mature bone-tendon junctions comprise tendon terminations, tendon junction regions, and calcified surfaces [159]. This necessitates the design of HFs with highly organized hierarchical structures for effective skeletal muscle repair. Additionally, the characteristics of presently developed HFs for *in vitro* assessment may not accurately simulate the dynamic *in vivo* environment [160]. These features change due to material degradation and tissue growth following implantation. Thus, the design and fabrication of 3D HFs with functional structure and performance should consider the dynamic alterations of the *in vivo* microenvironment. For example, the dynamic responsive scaffolds, such as pH-responsive viscoelastic HFs, should be developed to enable immediate restoration to their original positions and repair defects through instantaneous reassembly even after long-term exposure to various high strains [161]. Additionally, overcoming the considerable variability and scalability of injured muscle geometries is another crucial challenge that requires attention.

## 5 Conclusions and future directions

HFs, which mimic the properties of targeted tissue ECM, have emerged as a promising and effective approach for *in situ* soft tissue repair and regeneration by harnessing the regenerative potential of the human body. This review firstly introduces the concept and development of HFs, and summarizes their biomimetic properties in terms of hydrogels and micro-/nanofibers (Section 2). Afterward, the review discusses key requirements for the preparation of HFs, including raw material selection and process preparation, to serve as prognostic markers for future generations of such biomaterials (Section 3). When HFs are implanted *in vivo*, host cells adhere to the biomaterials and form local adhesions, enabling the identification of biochemical signals and physical cues from the biomaterial. These complex extracellular signal arrays, in turn, trigger intracellular signaling cascades and influence cell responses for tissue remodeling. Given the beneficial effects of biomimetic features and the significant advancements achieved, the review concludes with an overview of the research progress involving HFs in the regeneration and repair of the nerve, skin, cardiovascular system, and skeletal muscle (Section 4).

Despite significant advancements in recent years, HFs still exhibit certain limitations that warrant attention in future research. To achieve satisfactory outcomes for *in situ* tissue regeneration, HFs must closely replicate the natural tissue ECM in terms of physical attributes (e.g., mechanics, topology), chemical composition (regulation of functional groups), and biological cues (biological signaling). Such targeted designs are essential to provide supportive properties for cell growth and differentiation, facilitate cell-matrix interactions for tissue regeneration, and ensure a degradation rate that matches tissue remodeling: I) Physical Enhancements: these are primarily accomplished through fiber preparation and the construction of 3D structures. During fiber preparation, mechanical properties can be tailored by adjusting manufacturing parameters (e.g., crosslinking density, crosslinking time, hydrogel concentration), employing post-processing techniques (e.g., uniaxial tension), or integrating with support materials to form layered or core-shell composite structures. In 3D structure construction, scaffolds not only offer biochemical and biophysical stimulation and shield cells from harsh environments but also promote cell infiltration for complex tissue regeneration. However, the design of 3D HFs remains in its infancy, and the physicochemical properties of tissues regenerated using HFs often differ markedly from those of human skin. This highlights the need for substantial future advancements; II) Chemical Integration: a key challenge lies in incorporating multiple functions into a single hydrogel without compromising individual functionalities. For example, given the dynamic nature of the *in vivo* microenvironment, stimulus-responsive HFs (e.g., glucose, temperature, pH, ROS, and enzyme response) should be fabricated to adapt to the evolving microenvironment during tissue remodeling. HFs are well-suited for preparing sensors for personalized wound management, so smart HFs can be engineered as strain sensors to monitor the wound status and healing progress.

Additionally, because native tissues/organs exhibit multiscale hierarchical structures, multifunctional HFs should be designed to replicate these complex architectures, particularly key appendages such as blood vessels, hair follicles, or nerves, to support tissue healing and regeneration; III) Biological Cues: in addition to physical loading of biomolecules, reversible biochemical signals can be introduced into HFs to enable dynamic and spatiotemporal modulation of cell behaviors and tissue remodeling. Molecular biological approaches should be employed to elucidate further the pathophysiological processes involved in tissue regeneration. By understanding precise molecular mechanisms, HF development can be directed toward enhancing therapeutic outcomes. Furthermore, since most biomedical applications of HFs are currently confined to laboratory and animal model studies, extensive work remains to translate these findings into clinical trials, including comprehensive assessment of long-term toxicity to establish overall biosafety for human use, simplification of formulation strategies to enable efficient and reproducible large-scale manufacturing, and optimization of regulatory frameworks governing good manufacturing practices, quality control, safety standards, and patient protection.

Looking ahead, continuing to evolve HFs from passive scaffolds into active, instructive platforms will be essential to unlock the body's regenerative capabilities fully. This involves incorporating biofunctional cues that direct host cell recruitment and tissue-specific remodeling, alongside designs that encourage vascularization and innervation. Such approaches, combined with thorough preclinical validation and scalable manufacturing, will be vital to establishing HFs as genuine in-situ regenerative therapies.

## CRediT authorship contribution statement

**Bingcheng Yi:** Writing – original draft, Methodology, Investigation, Formal analysis, Conceptualization. **Xiaoyu Wang:** Writing – original draft, Methodology, Investigation, Formal analysis, Conceptualization. **Jiajia Yu:** Writing – original draft, Methodology, Investigation, Formal analysis. **Jiale Diao:** Writing – original draft, Methodology, Investigation. **Guangjun Wang:** Writing – original draft, Methodology, Investigation. **Shuo Li:** Writing – original draft, Methodology, Investigation. **Jiayi Bo:** Writing – original draft, Methodology, Investigation. **Xuemei Zhang:** Writing – original draft, Methodology, Investigation. **Chunling Zhang:** Writing – original draft, Methodology, Investigation. **Carlos F. Guimarães:** Writing – review & editing, Writing – original draft, Validation, Investigation, Conceptualization. **Qihui Zhou:** Writing – review & editing, Writing – original draft, Supervision, Resources, Methodology, Investigation, Funding acquisition, Formal analysis, Conceptualization. **Rui L. Reis:** Writing – review & editing, Supervision, Project administration, Funding acquisition.

## Ethics approval and consent

Not applicable.

## Declaration of competing interest

Rui L Reis is an associate editor for Bioactive Materials and was not involved in the editorial review or the decision to publish this article.

All authors declare that there are no competing interests.
